# Lipolytic efficacy of alginate double-layer nanoemulsion containing oleoresin capsicum in differentiated 3T3-L1 adipocytes

**DOI:** 10.1080/16546628.2017.1339553

**Published:** 2017-06-29

**Authors:** Mak-Soon Lee, Sunyoon Jung, Yoonjin Shin, Seohyun Lee, Chong-Tai Kim, In-Hwan Kim, Yangha Kim

**Affiliations:** ^a^ Department of Nutritional Science and Food Management, Ewha Womans University, Seoul, Republic of Korea; ^b^ Research Group of Bioprocess Engineering, Korea Food Research Institute, Seongnam, Republic of Korea; ^c^ Department of Food and Nutrition, Korea University, Seoul, Republic of Korea

**Keywords:** Free fatty acid, glycerol, lipolysis, mRNA expression, obesity

## Abstract

**Background**: Oleoresin capsicum (OC) is an organic extract from fruits of the genus *Capsicum*, and has been reported to have an anti-obesity effect.

**Objective**: This study comparatively investigated lipolytic effects of single-layer nanoemulsion (SN) and alginate double-layer nanoemulsion (AN) containing OC in 3T3-L1 adipocytes.

**Methods**: SN and AN were compared by analyzing the intracellular lipid accumulation, triglyceride (TG) content, release of free fatty acids (FFAs) and glycerol, and mRNA expression of genes related to adipogenesis and lipolysis were analyzed in fully differentiated 3T3-L1 adipocytes.

**Results**: Compared with SN, AN exhibited higher efficiency in inhibiting the intracellular lipid accumulation and TG content, and enhanced the release of FFAs and glycerol into the medium. In AN-treated cells, mRNA levels of peroxisome proliferator-activated receptor-γ and the fatty acid-binding protein adipocyte protein-2, which are involved in adipogenesis, were down-regulated, whereas those of genes related to lipolysis, including hormone-sensitive lipase and carnitine palmitoyl transferase-1α, were up-regulated compared with SN-treated cells.

**Conclusion**: The lipolytic effect of AN was greater than that of SN; this was partly associated with the increased TG hydrolysis via induction of lipolytic gene expression and suppression of adipogenic gene expression in 3T3-L1 adipocytes.​​​​

## Introduction

Adipocytes play an important role in energy homeostasis. In a condition of energy excess, adipocytes store surplus energy in the form of triglyceride (TG). within the cell, which can lead to obesity [1]. Given that obesity is highly associated with several metabolic diseases such as diabetes mellitus, cardiovascular diseases, and certain types of cancers [2], it is important for researchers to develop new compounds that effectively catabolize lipids accumulated in the adipocytes.

Oleoresin capsicum (OC) is an organic solvent extract from dried ripe fruits of the genus *Capsicum* [3]. It is widely used as a food additive to enhance the taste and shelf-life of food products [4]. Capsaicinoids contribute to the pungent flavor of the OC, and capsaicin is the main secondary metabolite in capsaicinoid present in red pepper [5]. The red pepper extract has shown beneficial effects on cancer [6], oxidative stress [7], and inflammation [7]. Furthermore, it has been shown that red pepper extract and capsaicin have anti-obesity effects *in vitro* [1,8] and *in vivo* [9,10]. In particular, an ethanol extract of red pepper seed was found to decrease glycerol-3-phosphate dehydrogenase (GPDH) activity and CCAAT-enhancer-binding protein-α (C/EBP)-α and C/EBP-β messenger RNA (mRNA) expression in 3T3-L1 adipocytes [11]. *Capsicum annuum* L. water extracts were found to inhibit lipoprotein lipase (LPL) activity and mRNA expression in 3T3-L1 cells [8]. In addition, capsaicin reduced the body weight gain and visceral adipose tissue mass in mice fed a high-fat diet [9] and decreased the fasting glucose/insulin and TG levels in the plasma of genetically obese/diabetic mice [10].

Nanoemulsions are oil-in-water emulsions with mean droplet diameters ranging from 20 to 500 nm [12]. Nanoemulsion technology has been successfully used in the food industry to encapsulate food components such as capsaicin and resveratrol in nanometer-sized structures [13]. The potential benefits of nanoemulsions include enhanced stability and oral bioavailability of hydrophobic functional food ingredients [14]. Choi et al. developed an OC-loaded double-layered nanoemulsion using a self-assembly emulsification method [13]. The stability and physicochemical properties of the double-layer nanoemulsion were enhanced compared with those of single-layered nanoemulsions, which implies that the double-layered nanoemulsion can be used in the production of functional foods containing lipophilic bioactive ingredients. Previously, we conducted an *in vivo* experiment by comparing the anti-obesity effects of OC and nanoemulsion oleoresin capsicum (NOC) [15]. NOC was found to be more effective than OC in inhibiting body weight gain and adipogenic gene expression in rats fed a high-fat diet. However, the detailed mechanism of the lipolytic effect of NOC involved in the anti-obesity effect remains unclear. In addition, studies comparing the lipolytic efficiency of the both single- and double-layered nanoemulsions have not been reported.

**Figure 1.**
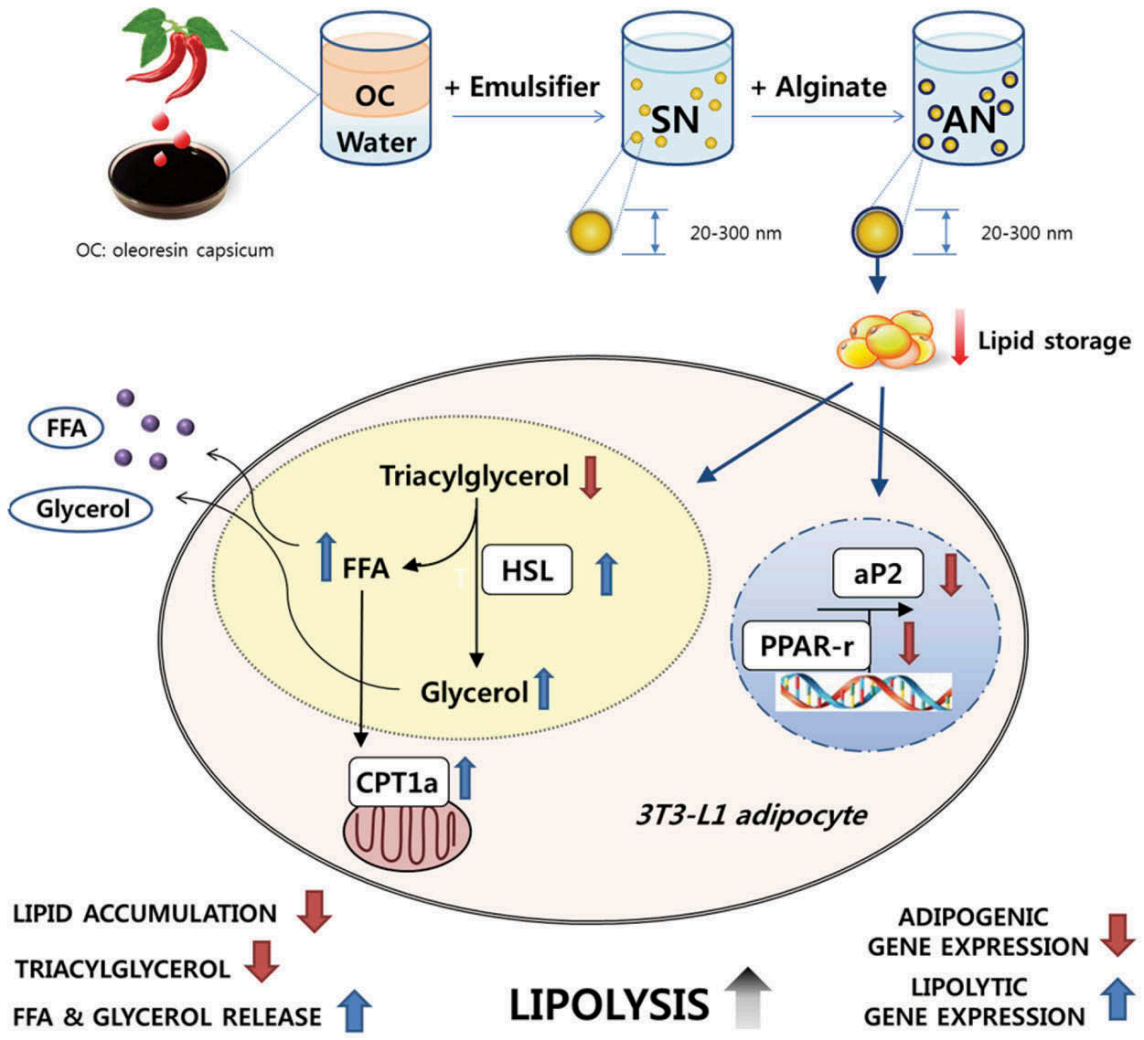
Schematic representation of a hypothesis on the efficacy of alginate double-layer nanoemulsion (AN) in 3T3-L1 adipocytes. OC, oleoresin capsicum; SN, single-layer nanoemulsion; FFA, free fatty acid; HSL, hormone-sensitive lipase; CPT1a, carnitine palmitoyl transferase-1α; aP2, adipocyte protein-2; PPAR-r, peroxisome proliferator-activated receptor-γ.

We hypothesized that a double-layer nanoemulsion containing OC (AN), which is coated with a natural biopolymer alginate, could have greater efficacy on lipolysis in 3T3-L1 adipocytes compared with a single-layer nanoemulsion containing OC (SN), as schematically presented in Figure 1. Therefore, we evaluated intracellular lipid accumulation and TG level, and the release of free fatty acids (FFAs) and glycerol under SN or AN treatment in 3T3-L1 adipocytes. In addition, the expression of genes related to adipogenesis, such as peroxisome proliferator-activated receptor-γ (PPAR-γ) and the fatty acid-binding protein adipocyte protein-2 (aP2), was compared. The mRNA levels of hormone-sensitive lipase (HSL) and carnitine palmitoyltransferase-1α (CPT-1α), involved in lipolysis, were also compared.

## Materials and methods

### Cells and reagents

The 3T3-L1 cell line was obtained from the American Type Culture Collection (Manassas, VA, USA). OC was supplied by General Foods and Flavors (Seoul, Korea). A 0.45 μm polyvinylidene fluoride (PVDF) membrane filter was purchased from Whatman (Maidstone, UK). Dulbecco’s modified Eagle’s medium (DMEM), glutamine, penicillin–streptomycin, fetal bovine serum (FBS), and TRIzol reagent were obtained from Invitrogen (Carlsbad, CA, USA). A cell count kit-8 (CCK-8) was purchased from Dojindo Laboratories (Kumamoto, Japan). Nonidet P-40 was purchased from Sigma-Aldrich (Saint Louis, MO, USA). Assay kits for TG were purchased from Asan Pharmaceuticals (Seoul, Korea). Moloney murine leukemia virus (M-MLV) reverse transcriptase was purchased from Promega (Madison, WI, USA). Universal SYBR® Green Master mix for quantitative polymerase chain reaction (qPCR) was purchased from Qiagen (Chatsworth, CA, USA). Assay kits for non-esterified FFAs and glycerol were obtained from Wako (Osaka, Japan) and Roche Molecular Biochemicals (Mannheim, Germany), respectively. A bicinchoninic acid (BCA) protein assay kit was obtained from Thermo Scientific (Pittsburgh, PA, USA).

### Preparation of SN and AN

Samples (SN and AN) were kindly supplied by the Korea Food Research Institute (Seongnam, Gyeonggi, Korea) [13]. Nanoemulsions were prepared using the self-assembly method and classified into two groups: SN based on the aqueous phase with water and AN based on alginate. In brief, the SN and AN were prepared using Tween 80, which was selected as the optimum detergent for the formation of oil-in-water (O/W) NOC by a pseudoternary phase diagram. The OC and Tween 80 (0.5 g) were mixed in the ratio of 1:3. The mixture was dissolved at 25°C for 2 h with constant stirring in 100 mL of water (SN) or alginate (0.5%, w/v) solution (AN) and then filtered using a 0.45 μm PVDF membrane filter. The filtrates were stabilized for 24 h at 25°C. The amounts of capsaicin in the SN and AN were quantified by high-performance liquid chromatography, and were prepared using the same concentration (5.1 μg/g).

### Cell culture and differentiation

The 3T3-L1 fibroblasts were maintained initially in DMEM supplemented with 10% (v/v) FBS, 2 mmol/L glutamine, 100 units/mL penicillin, and 100 μg/mL streptomycin at 37°C and 5% carbon dioxide. To induce adipocyte differentiation, 3T3-L1 cells were grown to confluence and cultured with a differentiation medium containing 0.5 mmol/L 3-isobutyl-1-methylxanthine, 1 μmol/L dexamethasone, and 5 μg/mL insulin (MDI). After 48 h of exposure to the differentiation medium, the cells were maintained for an additional 7 days in DMEM supplemented with 10% FBS. Fully differentiated 3T3-L1 adipocytes in serum-free medium containing 2% bovine serum albumin (BSA) were treated with indicated concentrations of SN or AN for 24 h. All the measurements were performed in triplicate.

### Cytotoxicity assay

Cell viability was determined as described previously [16] using a CCK-8 kit according to the manufacturer’s instructions. Differentiated 3T3-L1 adipocytes were treated with 0 (control), 0.1, 1, 10, 100, or 1000 ng/mL of SN or AN for 4 and 24 h at 37°C. For the negative and positive control samples, 0.1% dextran and 0.1% sodium dodecyl sulfate (SDS) was added, respectively. Absorbance was measured using a Varioskan plate reader at 450 nm (Thermo Electron, Waltham, MA, USA) and results are presented as the percentage of untreated control cells. All measurements were performed in at least three independent experiments, each of which was performed in triplicate (*n* = 3).

### Oil Red O staining

Lipid accumulation was measured as previously described [16]. In brief, fully differentiated 3T3-L1 adipocytes were maintained in serum-free medium containing 2% BSA with 0, 1, 10, 100, or 1000 ng/mL of SN or AN for 24 h. The cells were washed with phosphate-buffered saline (PBS; pH 7.4) and fixed with 10% (v/v) formalin in PBS. The accumulation of lipid was measured by staining the cells with Oil Red O (saturated Oil Red O dye in six parts isopropanol and four parts water) for 15 min. Quantification of the stain was carried out by dissolving the stained oil droplets in the cell monolayer with 4% (v/v) Nonidet P-40 in isopropanol, followed by measuring the absorbance at 520 nm. Values are presented as the percentage of control cells treated without SN or AN.

### TG assay

For the measurement of intracellular TG, a TG assay kit was used according to the method described previously [16]. Differentiated 3T3-L1 adipocytes were lysed in a lysis buffer that consisted of 1% Triton X-100 in PBS, and the cellular content of TG was determined using a commercial TG assay kit. The cellular TG content was then normalized to the protein concentration measured by a BCA protein assay kit.

### FFA and glycerol assay

To investigate the concentration-dependent effects, differentiated 3T3-L1 adipocytes were treated in serum-free medium containing 2% BSA with 0 (control), 1, 10, 100, or 1000 ng/mL of SN or AN for 24 h. To investigate the concentration-dependent effects, 1000 ng/mL of SN or AN was treated for 1, 3, 6, 16, and 24 h. Medium was collected from the culture plate and heated at 65°C for 8 min to inactivate any enzymes released from the cells. The amounts of FFAs and glycerol released into the medium were measured using colorimetric enzyme assay kits in accordance with the manufacturer’s instructions. The cellular protein concentration was determined using a BCA protein assay kit. The glycerol and FFA concentrations were normalized to the cellular protein content.

### Real-time qPCR

Total RNA was extracted from 3T3-L1 adipocytes using TRIzol Reagent. Complementary DNA (cDNA) was synthesized from 4 μg RNA using M-MLV reverse transcriptase. qPCR was then performed in 25 μL Universal SYBR Green Master mix using a fluorometric thermal cycler (Rotor-Gene^TM^ 2000; Corbett Research, Mortlake, NSW, Australia). The reaction mixtures were incubated for initial denaturation at 95°C for 15 min, followed by 50 cycles of PCR. Each cycle included denaturation at 95°C for 15 s, annealing at 55°C for 20 s, and primer extension at 72°C for 20 s. Primers were designed using an online program (primer3_http://www.cgivo.2) [17]. Sequences of the sense and antisense primers used for amplification were: PPAR-γ, 5′-TGTGGGGATAAAGCATCAGC-3′ and 5′-CAAGGCACTTCTGAAACCGA-3′; aP2, 5′-TCACCCCAGATGACAGGAAA-3′ and 5′-CATGACACATTCCACCACCA-3′; HSL, 5′-ACTCAGACCAGAAGGCACTA-3′ and 5′-TAGTTCCAGGAAGGAGTTGA-3′; CPT-1α, 5′-CTGTTGGAGGTGACAGACTT-3′ and 5′-CACTTTCTCTTTCCACAAGG-3′; and β-actin, 5′-GTTGCCAATAGTGATGACCT-3′ and 5′-GGACCTGACAGACTACCTCA-3′. The ^ΔΔC^_T_ method was used for relative quantification, and its value for each sample was determined by calculating the difference between the C_T_ value of the target gene and the C_T_ value of the β-actin reference gene. The normalized level of expression of the target gene in each sample was calculated using the formula 2^−ΔΔC^_T_. Values were expressed as a fold of the control.

### Statistical analysis

Values are expressed as mean ± SE. Statistical analyses were performed using SPSS software (version 19; IBM Corp., Armonk, NY, USA). The significance of differences between two groups of SN and AN at the same concentration were determined with a Student’s *t* test (two-tailed). Significant differences among different concentrations of treatment group were analyzed using a one-way analysis of variance (ANOVA), followed by Tukey’s multiple comparison tests. A value of *p* < 0.05 indicated a significant difference.

## Results

### Effects of SN and AN on viability of 3T3-L1 adipocytes

Potential cytotoxic effects of SN and AN on 3T3-L1 adipocytes were investigated. 3T3-L1 adipocytes were treated with 0 (control), 0.1, 1, 10, 100, and 1000 ng/mL SN or AN, and incubated for 4 or 24 h. Cell viability of SN, AN, or 0.1% dextran negative control (NC) remained unchanged at various concentrations after 4 and 24 h of incubation (supplementary Figure S1), and the optical density (OD) ranged from 0.83 to 1.30. However, in the positive control (PC) with 0.1% SDS, cell viability was quite apparently decreased by 93.1–93.7% (0.03–0.08 OD) after 4 h of incubation, compared to untreated control.

### Effects of SN and AN on intracellular lipid accumulation and TG content in 3T3-L1 adipocytes

**Figure 2.**
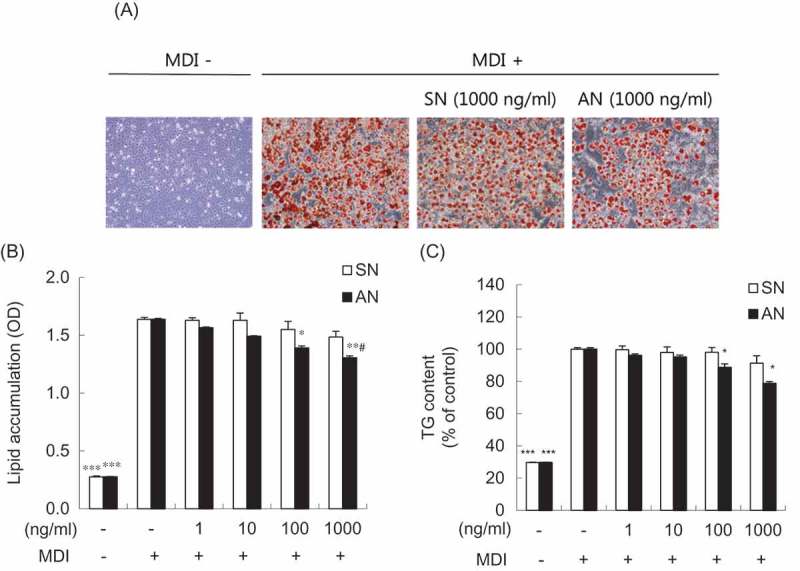
Effects of single-layer nanoemulsion (SN) and alginate double-layer nanoemulsion (AN) on the lipid accumulation and triglyceride (TG) content in 3T3-L1 adipocytes. (a) The effect of SN and AN on lipid droplet formation was measured by Oil Red O staining. Differentiated 3T3-L1 adipocytes were treated with 1000 ng/mL of SN or AN for 24 h. Representative cell images were captured at 100× magnification. MDI, differentiation medium containing 3-isobutyl-1-methylxanthine, dexamethasone, and insulin. Quantification of (b) intracellular lipid accumulation and (c) TG content. Differentiated 3T3-L1 adipocytes were treated with 0 (MDI treated control), 1, 10, 100, or 1000 ng/mL of SN or AN for 24 h. Oil Red O-stained lipids were extracted in absolute isopropanol, after which the absorbance of the solution was measured at 520 nm. TG content was determined using enzymic colorimetric methods. OD, optical density. Data are expressed as the mean ± SE of at least three independent experiments, each performed in triplicate (*n* = 3). One-way ANOVA followed by Tukey’s multiple comparison tests: *p < 0.05, **p < 0.01, and ***p < 0.001 compared with MDI treated control group. Student’s *t* test: #*p* < 0.05 compared with SN group.​​​​

To evaluate the effects of SN and AN on intracellular lipid accumulation and TG contents, fully differentiated adipocytes were treated with 0 (control), 1, 10, 100, and 1000 ng/mL of SN or AN for 24 h. The intracellular lipid content decreased in a concentration-dependent manner by 15% and 20% in 100 and 1000 ng/mL of AN, respectively, compared with that of the control (Figure 2(b)). Furthermore, the intracellular lipid content of AN was found to be lower than that of SN by 12.1% in 1000 ng/mL (Figure 2(a,b)). The TG content decreased by 11% and 21% in 100 and 1000 ng/mL of AN, respectively, compared with that of the control (Figure 2(c)). On the other hand, there were no significant differences in the presence of SN (Figure 2(c)).

### Effects of SN and AN on FFA and glycerol concentration in 3T3-L1 adipocytes

**Figure 3.**
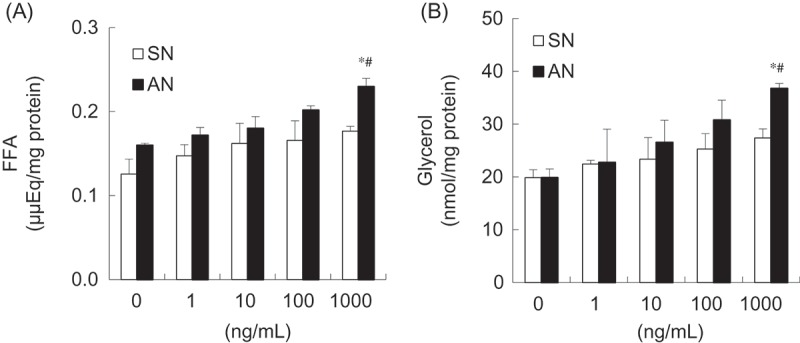
Concentration-dependent effects of single-layer nanoemulsion (SN) and alginate double-layer nanoemulsion (AN) on (a) free fatty acids (FFA) and (b) glycerol from adipocytes. Differentiated 3T3-L1 adipocytes were treated with 0 (untreated control), 1, 10, 100, or 1000 ng/mL of SN or AN for 24 h. The culture medium was collected and assayed for the FFA and glycerol content. Data are expressed as the mean ± SE of at least three independent experiments, each performed in triplicate (*n* = 3). One-way ANOVA followed by Tukey’s multiple comparison tests: *p < 0.05 compared with untreated control group. Student’s *t* test: #*p* < 0.05 compared with SN group.

**Figure 4.**
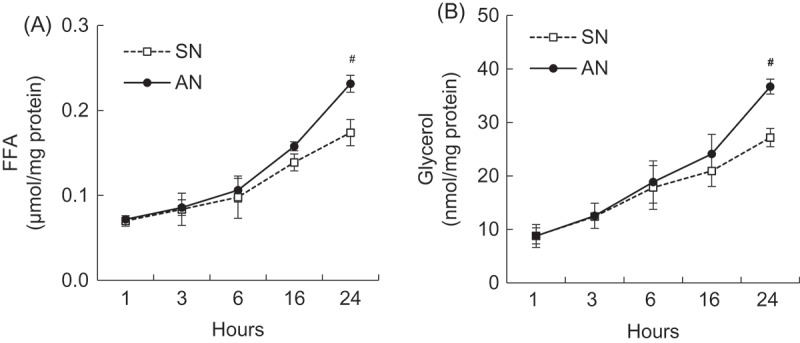
Time-dependent effects of single-layer nanoemulsion (SN) and alginate double-layer nanoemulsion (AN) on (a) free fatty acids (FFA) and (b) glycerol from adipocytes. Differentiated 3T3-L1 adipocytes were treated with 1000 ng/mL of SN or AN for 1, 3, 6, 16, or 24 h. The culture medium was collected and assayed for the FFA and glycerol content. Data are expressed as the mean ± SE of at least three independent experiments, each performed in triplicate (*n* = 3). Student’s *t *test: #*p* < 0.05 compared with SN group.

Lipolytic effects of the SN and AN on fat disintegration were measured by estimating the amounts of FFA and glycerol released in the medium of adipocytes. The effect of SN and AN on FFA and glycerol was both concentration and time dependent. First, to investigate the concentration-dependent effects, the differentiated 3T3-L1 adipocytes were treated with various concentrations [0 (control), 0.1, 1, 10, 100, and 1000 ng/mL] of SN and AN for 24 h. AN significantly increased the amount of FFA and glycerol released into the medium at 1000 ng/mL, by 30% and 45%, respectively, compared with the control. However, there were no significant differences in the presence of SN (Figure 3). In addition, the amounts of FFA and glycerol were significantly higher (by 23% and 26%, respectively) in the AN-treated than in the SN-treated 3T3-L1 adipocytes (Figure 3). To investigate the time-dependent effects, we treated the differentiated 3T3-L1 adipocytes with 1000 ng/mL of SN or AN for 1, 3, 6, 16, and 24 h. The amounts of FFA and glycerol tended to increase over time with the treatment of SN and AN. Specifically, after 24 h of incubation, the amounts of FFA and glycerol released into the medium were higher by 24.9% and 26.0%, respectively, when treated with AN than with SN (Figure 4).

### Effects of SN and AN on gene expression in 3T3-L1 adipocytes

**Figure 5.**
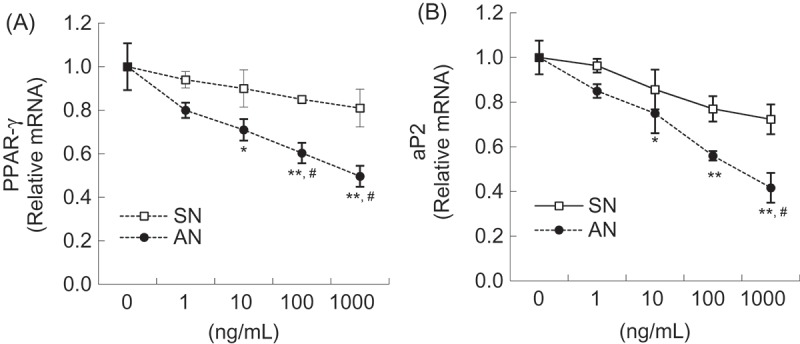
Effects of single-layer nanoemulsion (SN) and alginate double-layer nanoemulsion (AN) on the mRNA expression of adipogenic genes including (a) peroxisome proliferator-activated receptor-γ (PPAR-γ) and (b) adipocyte protein-2 (aP2) in adipocytes. Differentiated 3T3-L1 adipocytes were treated with 0 (untreated control), 1, 10, 100, or 1000 ng/mL of SN or AN for 24 h. The mRNA level was measured using quantitative polymerase chain reaction. The values were calculated as fold-change over the control. Data are expressed as the mean ± SE of at least three independent experiments, each performed in triplicate (*n* = 3). One-way ANOVA followed by Tukey’s multiple comparison tests: *p < 0.05 and **p < 0.01 compared with untreated control group. Student’s *t* test: #*p* < 0.05 compared with SN group.

**Figure 6.**
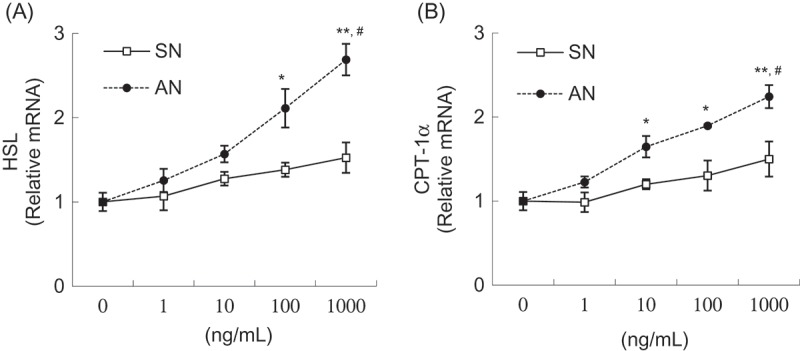
Effects of single-layer nanoemulsion (SN) and alginate double-layer nanoemulsion (AN) on the mRNA expression of lipolytic genes including (a) hormone-sensitive lipase (HSL) and (b) carnitine palmitoyl transferase-1α (CPT-1α) in adipocytes. Differentiated 3T3-L1 adipocytes were treated with 0 (untreated control), 1, 10, 100, or 1000 ng/mL of SN or AN for 24 h. The mRNA level was measured using quantitative polymerase chain reaction. The values were calculated as fold-change over the control. Data are expressed as the mean ± SE of at least three independent experiments, each performed in triplicate (*n* = 3). One-way ANOVA followed by Tukey’s multiple comparison tests: *p < 0.05 and **p < 0.01 compared with untreated control group. Student’s *t* test: #*p* < 0.05 compared with SN group.

We evaluated whether SN or AN stimulates lipid catabolism by regulating genes involved in adipocyte differentiation and lipolysis. AN was found to down-regulate mRNA levels of adipogenic genes such as PPAR-γ and aP2 in a concentration-dependent manner compared with those of the control; however, SN did not exhibit any such effect (Figure 5). The mRNA levels of PPAR-γ and aP2 were significantly lower at 1000 ng/mL of AN than SN, by 38.7% and 42.4%, respectively (Figure 5). The mRNA levels of lipolytic genes such as HSL and CPT-1α were up-regulated by AN in a concentration-dependent manner compared with those of the control; however, no such effect was shown by SN (Figure 6). Specifically, the mRNA levels of HSL and CPT-1α were significantly higher at 1000 ng/mL of AN than SN, by 43.2% and 33.1%, respectively (Figure 6).

## Discussion

Food scientists have been striving to discover better food products with health benefits. Nanoemulsion delivery systems have been known to enhance the bioavailability of insoluble functional food ingredients [14]. In a previous study, Choi et al. established optimal conditions for the production of a stable nanoemulsion containing OC and developed SN and AN, which were all used in our study [13,18]. The SN is a single-layer nanoemulsion including OC, while the AN is a double-layer nanoemulsion developed to increase the stability and physicochemical properties of the SN. The AN showed a more compact and clearer particle structure than the SN, and was found to have higher zeta potential value, which is an indicator of the greater overall stability of the nanoemulsion compared to the SN [13]. In a 2014 study, we reported that NOCs have beneficial effects against obesity compared with OC in reducing fat mass and adipogenic gene expression in obese rats fed a high-fat diet [15]. In this study, we focused on the lipolytic effects of SN and AN in differentiated 3T3-L1 adipocytes.

To evaluate the inhibitory effects of the SN and AN on fat accumulation, the content of intracellular lipid and TG in 3T3-L1 adipocytes was measured. The current study showed that AN decreased intracellular lipid and TG, in a concentration-dependent manner. This result was consistent with our previous study reporting that NOC had anti-obesity effects that lowered white adipose tissue mass in rats fed a high-fat diet [15]. Here, we compared the bioefficacy of two types of NOC (SN and AN), and demonstrated that AN may be a more effective material to lower lipid accumulation than SN. Because a double-layer nanoemulsion incorporated with alginate has been reported to improve the stability of a single-layer nanoemulsion [13], we assume that improved stability of the bioactive ingredients in AN, compared with SN, may enhance the bioefficacy of the AN formulation.

In lipolysis, TG is broken down into FFAs and glycerol. To estimate the direct effect of SN and AN on lipolysis, the amount of FFAs and glycerol released into the medium was measured in 3T3-L1 adipocytes. In our previous study, capsaicin was shown to increase the release of FFAs and glycerol [16]. Here, AN tended to enhance the release of FFAs and glycerol in a concentration- and time-dependent manner; however, such an effect was not exhibited by SN. Furthermore, AN was more effective than SN in stimulating fat hydrolysis in mature adipocytes, as shown by higher amounts of FFAs and glycerol released after AN treatment than SN treatment. Since there were no data focusing on lipolytic effects of the NOC, this study suggests possible mechanisms for explaining the anti-obesity effect of NOC. In addition, it can be assumed that AN may have better lipolytic effects than SN, which were modulated by enhancing the hydrolysis of TG into glycerol and FFAs in adipocytes.

Adipogenesis is the process by which preadipocytes become mature adipocytes. The differentiation and proliferation of preadipocytes are regulated and coordinated by several adipogenic molecules, such as PPAR-γ and aP2 [19]. PPAR-γ is a key transcriptional factor expressed at high levels in adipocytes [20]. When the expression of PPAR-γ is activated, lipid biosynthesis pathways are stimulated through target gene expression such as C/EBP-α and aP2 [19,21]. The latter (aP2) is a carrier protein for fatty acids and is highly expressed during adipocyte differentiation [22]. Circulating aP2 levels increase in both genetically and diet-induced obese mice, and reducing the circulating aP2 levels to normal improves metabolic parameters in obese mice [23]. Feng et al. reported that yellow capsicum extract inhibits the expression of PPAR-γ and C/EBP-α in 3T3-L1 adipocytes [24]. Furthermore, OC and NOC extracts were reported to decrease the mRNA levels of adipogenic genes including PPAR-γ, sterol regulatory element binding protein-1c (SREBP-1c), and aP2 in white adipose tissue of obese rats fed a high-fat diet [15]. In this study, we found that AN reduced mRNA levels of PPAR-γ and aP2 in a concentration-dependent manner. Specifically, AN significantly lowered mRNA levels of PPAR-γ and aP2 compared with SN, which implies greater efficacy of AN in inhibiting adipogenic gene expression.

To evaluate the lipolytic mechanisms of the SN and AN, the mRNA levels of CPT-1α and HSL were evaluated. HSL is a key enzyme for the regulation of lipid storage. Upon lipolytic stimulation, HSL is activated, which hydrolyzes triacylglycerol to monoacylglycerol and FFAs [25]. Fatty acyl-coenzyme A (CoA) is generated from FFAs in the cytoplasm by fatty acyl-CoA synthase. As a result, CPT-1α, located on the outer mitochondrial membrane, catalyzes the formation of fatty acyl–carnitine from fatty acyl-CoA and carnitine. The acyl–carnitine complexes are then transported across the inner mitochondrial membrane to be oxidized [26]. Therefore, CPT-1α serves as a rate-limiting enzyme for fatty acid oxidation and is indicative of lipolysis. Studies have demonstrated that red pepper and capsaicin are effective in inducing lipolysis by regulating genes involved in lipid catabolism [16,27]. Capsaicin up-regulates the expression of HSL, CPT-1α, and uncoupling protein-2 (UCP2) in adipocytes [16]. Besides, Kochujang (Korean fermented red pepper paste) extract up-regulates HSL transcriptional activity in 3T3-L1 adipocytes [27]. In our study, AN increased the expression of HSL and CPT-1α in a concentration-dependent manner; however, no such effect was exhibited by SN. Moreover, AN was more efficient than SN at increasing mRNA levels of HSL and CPT-1α. These results indicate that the lipolytic action of AN could be partly associated with the expression of genes related to lipolytic pathways, including HSL and CPT-1α.

In conclusion, the lipolytic activity of AN was found to be higher than that of SN, leading to reduced intercellular lipid and TG concentrations and increased hydrolysis of TG to release FFAs and glycerol into the medium of adipocytes. The lipolytic effects are mediated, at least in part, by regulation of the expression of genes involved in the lipolytic pathway, such as HSL and CPT-1α, and those involved in adipogenesis, such as PPAR-γ and aP2 expression in adipocytes. Thus, AN may be useful as a potent lipolytic agent for treating obesity.

## Supplementary Material

Supplementary_figure-ZFNR-2016-0064-r.docx
