# Supplementary material for: Lipolytic efficacy of alginate double-layer nanoemulsion containing oleoresin capsicum in differentiated 3T3-L1 adipocytes
Source: Food Nutr Res. 2017 Jun 29;61(1):1339553. doi: 10.1080/16546628.2017.1339553 (PMC5510203; doi:10.1080/16546628.2017.1339553)
Supplement: Supplementary_figure-ZFNR-2016-0064-r.docx [file zfnr_a_1339553_sm2729.docx]

**Supplementary material**

| (A) |
| --- |
|  |
| (B) |
|  |

***Fig S1.*** Effects of single-layer nanoemulsion (SN) and alginate double-layer nanoemulsion (AN) on the viability of 3T3-L1 adipocytes after 4 h (A) and 24 h (B) of incubation. 3T3-L1 cells were treated with 0 (untreated control), 0.1, 1, 10, 100, or 1000 ng/mL of SN or AN, and incubated for the times indicated. Cell viability was determined using a WST-8 assay. Data are expressed as mean ± SE (n = 3). Negative control (NC), 0.1% dextran; positive control (PC), 0.1% SDS. One-way ANOVA followed by Tukey’s multiple comparison tests: ^***^*p* < 0.001 compared with untreated control group.
